# Correction: Wang et al. Anti-Metabolic Syndrome Effects of Fucoidan from *Fucus vesiculosus* via Reactive Oxygen Species-Mediated Regulation of JNK, Akt, and AMPK Signaling. *Molecules* 2019, *24*, 3319

**DOI:** 10.3390/molecules30122574

**Published:** 2025-06-13

**Authors:** Xueliang Wang, Xindi Shan, Yunlou Dun, Chao Cai, Jiejie Hao, Guoyun Li, Kaiyun Cui, Guangli Yu

**Affiliations:** 1 Key Laboratory of Marine Drugs of Ministry of Education, Shandong Provincial Key Laboratory of Glycoscience and Glycotechnology, School of Medicine and Pharmacy, Ocean University of China, Qingdao 266003, China; 2 Laboratory for Marine Drugs and Bioproducts, Pilot National Laboratory for Marine Science and Technology (Qingdao), Qingdao 266237, China

In the original publication [1], the representative image of AKT (Figure 1e) and HMGCR (Figure 2b) were inadvertently misplaced. The corrected Figure 1 and Figure 2 are featured below. The authors apologize for any inconvenience caused and state that the scientific conclusions are unaffected. This correction was approved by the Academic Editor. The original publication has also been updated.

## Figures and Tables

**Figure 1:**
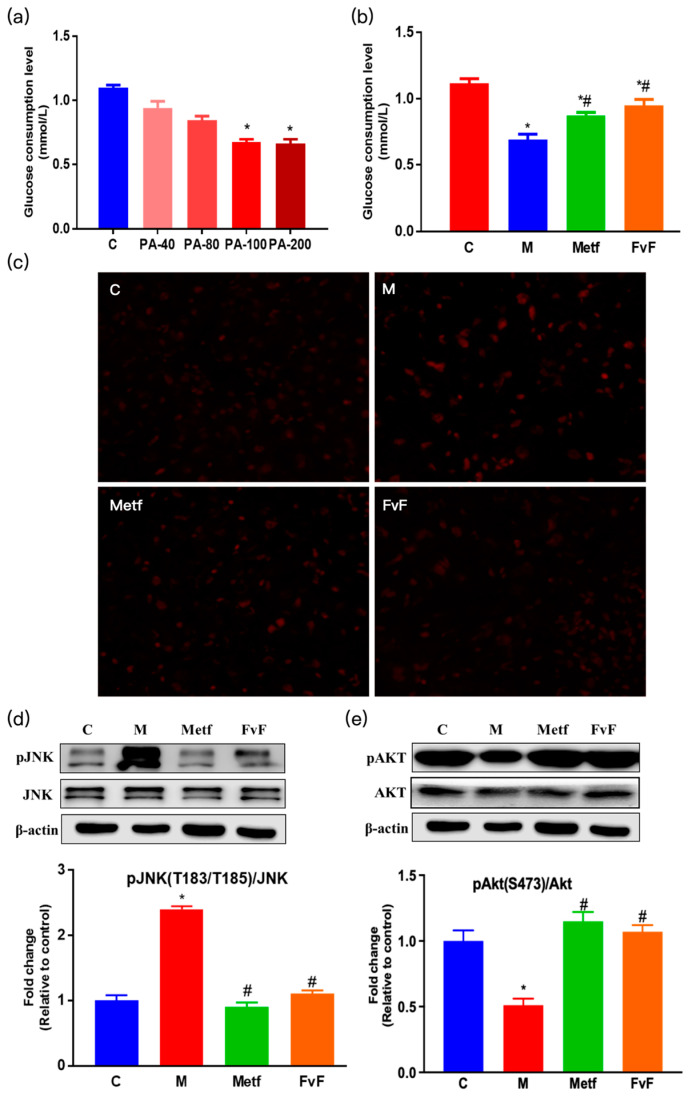
Effects of fucoidan from *Fucus vesiculosus* (FvF) on relieving insulin resistance (IR) in HepG2 cells. Effects of sodium palmitate (PA) on cellular glucose consumption (**a**). Cells were treated with a concentration range of PA for 24 h. Effects of FvF on glucose consumption in IR cells (**b**). Cells were treated with Metf (2 mM) or FvF (100 μg/mL) in the presence of 100 μM PA for 24 h (**c**). Reactive oxygen species (ROS) was detected by in situ dihydroethidium (DHE) staining (200×). C, control group; M, cells were treated with 100 μM PA for 24 h; Metf and FvF, cells were treated with metformin (2 mM) or FvF (100 μg/mL) in the presence of 100 μM PA for 24 h. Phosphorylation of c-Jun N-terminal kinase (pJNK) (**d**) and phosphorylation of protein kinase B (pAkt) (**e**); protein levels changed between different treatment groups. C, control group; M, cells treated with 100 μM PA for 24 h; Metf and FvF, cells treated with 100 μM PA for 24 h and then incubated with metformin (2 mM) or FvF (100 μg/mL) for another 6 h. Data are expressed as the mean ± SEM. Differences were assessed by ANOVAs and statistical results are denoted as follows: * *p* < 0.05 versus the control group; # *p* < 0.05 versus the model group.

**Figure 2:**
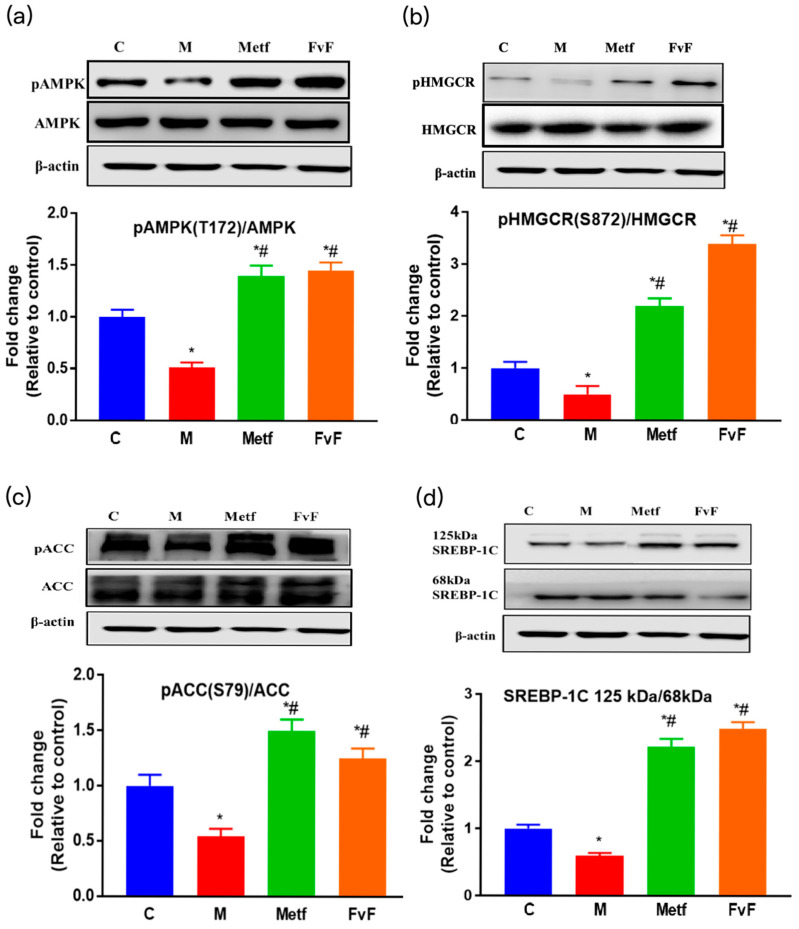
The effects of fucoidan from *Fucus vesiculosus* (FvF) on the adenosine 5’-monophosphateativated protein kinase (AMPK) signaling pathway. The effects of FvF on the regulation of pAMPK (**a**), phosphorylation of HMG-CoA reductase (pHMGCR) (**b**), phosphorylation of acetyl-CoA carboxylase (pACC) (**c**), and sterol-regulatory element-binding protein-1c (SREBP-1C) (**d**). C, control group; M, cells treated with 100 μM sodium palmitate (PA) for 24 h; Metf and FvF, cells treated with 100 μM PA for 24 h and then incubated with metformin (2 mM) or FvF (100 μg/mL) for another 6 h. SREBP-1C ratio was calculated as 125 kDa immature form divided by 68 kDa mature form. Data are expressed as the mean ± SEM. Differences were assessed by ANOVAs and statistical results are denoted as follows: * *p* < 0.05 versus the control group; # *p* < 0.05 versus the model group.
